# Effects of Physiological Internal Noise on Model Predictions of Concurrent Vowel Identification for Normal-Hearing Listeners

**DOI:** 10.1371/journal.pone.0149128

**Published:** 2016-02-11

**Authors:** Mark S. Hedrick, Il Joon Moon, Jihwan Woo, Jong Ho Won

**Affiliations:** 1 Department of Audiology and Speech Pathology, University of Tennessee Health Science Center, Knoxville, TN, United States of America; 2 Department of Otorhinolaryngology-Head and Neck Surgery, Samsung Medical Center, Sungkyunkwan University, School of Medicine, Seoul, Korea; 3 Department of Biomedical Engineering, University of Ulsan, Ulsan, Korea; University of Salamanca- Institute for Neuroscience of Castille and Leon and Medical School, SPAIN

## Abstract

Previous studies have shown that concurrent vowel identification improves with increasing temporal onset asynchrony of the vowels, even if the vowels have the same fundamental frequency. The current study investigated the possible underlying neural processing involved in concurrent vowel perception. The individual vowel stimuli from a previously published study were used as inputs for a phenomenological auditory-nerve (AN) model. Spectrotemporal representations of simulated neural excitation patterns were constructed (i.e., neurograms) and then matched quantitatively with the neurograms of the single vowels using the Neurogram Similarity Index Measure (NSIM). A novel computational decision model was used to predict concurrent vowel identification. To facilitate optimum matches between the model predictions and the behavioral human data, internal noise was added at either neurogram generation or neurogram matching using the NSIM procedure. The best fit to the behavioral data was achieved with a signal-to-noise ratio (SNR) of 8 dB for internal noise added at the neurogram but with a much smaller amount of internal noise (SNR of 60 dB) for internal noise added at the level of the NSIM computations. The results suggest that accurate modeling of concurrent vowel data from listeners with normal hearing may partly depend on internal noise and where internal noise is hypothesized to occur during the concurrent vowel identification process.

## Introduction

Human listeners often engage in conversation in an acoustic environment where the surrounding voices interfere with understanding the speech produced by the talker of interest. To address the “cocktail party problem” without visual cues, the auditory system has to identify, segregate, and group individual speech signals on the basis of the single temporal waveform that is formed as a result of the summation of concurrent speech signals (for review, see [1, 2]). To understand the potential psychoacoustic and neural mechanisms for speech understanding in background noise, the concurrent vowel identification paradigm has been widely used particularly with reference to competing talkers [3, 4, 5, 6]. Because vowels may be separated by differences in fundamental frequency (F0), models to explain identification of concurrent vowels have frequently focused on F0 segregation. There are, however, other cues which may be used to separate concurrent vowels. One such cue is temporal onsets/offsets [7, 8]. Previous research has shown that temporal asynchronous onsets can be efficiently used by listeners to separate and identify concurrent vowel stimuli [9, 10, 11, 12], even if both vowels have the same F0.

Many of the models that have been posited to explain concurrent vowel perception have focused either on comparison of cochlear excitation patterns [3, 11, 13, 14, 15], autocorrelation of auditory filter/inner hair cell channels [4], frequency and temporal resolution [14], or auditory-nerve discharge timing involving harmonic cancellation [6, 16, 17]. Other studies have posited that the sound source segregation needed to identify concurrent vowels likely involves multistage processing in both primary and association auditory cortices [18]. While it may be that primitive processing of acoustic cues such as F0 may provide robust cues for younger listeners with normal-hearing (NH) sensitivity [19, 20], it may be that other processes play an important role, particularly if F0 cues are unavailable [21]. Recent work to explain vowel confusion patterns using full F0-based segregation algorithms has shown inconsistencies in the ability of these algorithms to predict and mimic the behavioral data [22]. Thus, other factors besides F0 differences could potentially influence listeners’ identification of concurrent vowels; one or more of these factors may involve non-primitive sound segregation processes [12].

To help determine what other factors influence identification of concurrent vowels, and to test whether source segregation processes affect concurrent vowel identification, we selected a data set of concurrent vowel identification lacking in F0 cues but including a source segregation cue—that of temporal onset asynchrony [12]. We sought to determine the efficacy of computational modeling in simulating this data set from human NH listeners. Because previous models of concurrent vowel identification have suggested multistage processing including cochlear nerve function and sound source segregation [2, 18], our preliminary computational modeling included more than one scheme: a phenomenological auditory-nerve (AN) model [23] and a procedure for comparing simulated neural excitation patterns of the AN model to predict concurrent vowel identification performance. It was hypothesized that (1) simulated concurrent vowel identification scores would increase as a function of temporal onset asynchrony; and (2) further restriction of model computation may be needed to best match the human data.

## Materials and Methods

### Subjects

The concurrent vowel identification data for 14 normal-hearing listeners (mean age = 24 years; 12 females and 2 males) were adopted from previous work [12]. All listeners had audiometric thresholds ≤ 15 dB HL for the octave frequencies between 500 and 8000 Hz in each ear. All listeners were native speakers of American English. The study and the written informed consent procedure were approved by the University of Tennessee Institutional Review Board (IORG0000051).

### Stimuli

The vowel stimuli used in the current study were from a previous study [12]. The American English vowels /i ɑ u ӕ ɝ/ (S1–S5 Files) with a duration of 200 ms were synthesized using the cascade option of a software formant synthesizer [24] at a 10 kHz sampling rate. To test the effects of onset asynchrony without any contributing effects of F0, all five vowels had identical F0 of 120 Hz. The vowels were shaped using 10 ms cosine ramps to avoid production of transients. All five vowels were equalized to the same root-mean-square (RMS) level. Seven vowel pairs were then created: /u i/, /ӕ ɑ/, /ɝ ɑ/, /ɝ ӕ/, /ӕ i/, /ɝ i/, and /ɝ u/. These combinations were chosen because they showed the largest effect of onset asynchrony in the pilot study for previous work [12]. These vowel pairs were presented with temporal onset asynchronies of 0, 25, 50, 75, 100, 125, and 150 ms. Whenever a cardinal vowel (/i ɑ u/) was paired with another vowel, the cardinal vowel began after the other vowel. The vowel pairs were low-pass filtered at 4.8 kHz and routed to Sennheiser HD headphones located inside a double-walled IAC sound attenuating booth. The pair of concurrent vowels was presented to the listeners monaurally at 60 dB SPL. The same set of vowel stimuli was used for the current modeling study. Details of stimuli are described in [12], and the formant values of the stimuli are shown in Table 1.

**Table 1:** Formant frequency values for vowel stimuli.

Vowel	/e/	/a/	/u/	/ae/	/er/
IPA	/i/	/ɑ/	/u/	/æ/	/ɝ/
Supporting File	S1 File	S2 File	S3 File	S4 File	S5 File
F1	250	750	250	750	450
F2	2250	1050	850	1450	1150
F3	3350	2950	2250	2450	1250
F4	3350	3350	3350	3350	3350

### Human concurrent vowel identification test procedure

In previous work [12], the seven vowel pairs combined with the seven onset asynchrony values yielded a total of 49 stimuli. The list of 49 stimuli comprised one block. During each block, 49 stimuli were presented in random order. After hearing a mixture of concurrent vowels, listeners indicated the two individual vowels that they heard. For each testing block, an identification score was calculated after 49 concurrent vowel presentations as the percent of both vowels correctly identified. Each listener performed five blocks of identification tests and the mean identification scores averaged across the five testing blocks are reported as a function of onset asynchrony.

### Computational model of peripheral auditory processing

A phenomenological model of the AN [23] was used to simulate neural responses to the identical stimuli used in the human concurrent vowel identification task. The basic framework of this model has been tested extensively against animal physiological data and psychophysical data in response to both simple and complex stimuli, such as tones, broadband noise, and speech-like signals [25, 26, 27]. This model was chosen for the current study because it incorporates diverse nonlinear physiological properties of the cochlea, including compression, suppression, broadened tuning, and best-frequency shifts with increases in sound level. Inputs for the AN model were five single vowels or seven mixtures of concurrent vowels. The neural response at each characteristic frequency (CF) was created from the post stimulus time histogram (PSTH) of 50 simulated AN fibers. The PSTH at 30 CFs, spaced logarithmically between 100 and 5000 Hz, were then obtained. The outputs of the AN model were smoothed by convolving them with a 50% overlap, 128 sample Hamming window. In accordance with the neuroanatomical data observed in cats [28], 60% of the AN fibers were set to have a high spontaneous rate (> 18 spikes/sec), 20% medium (0.5–18 spikes/sec), and 20% low (< 0.5 spikes/sec). All model simulations were obtained with the intact functionality of inner and outer hair cells by setting the model to be the normal-hearing mode (i.e., C_IHC_ and C_OHC_ = 1.0). The input stimulus level was scaled to 60 dB SPL prior to presentation to the model, consistent with the human testing.

#### Neurogram Similarity Index Measure

**Fig 1:**
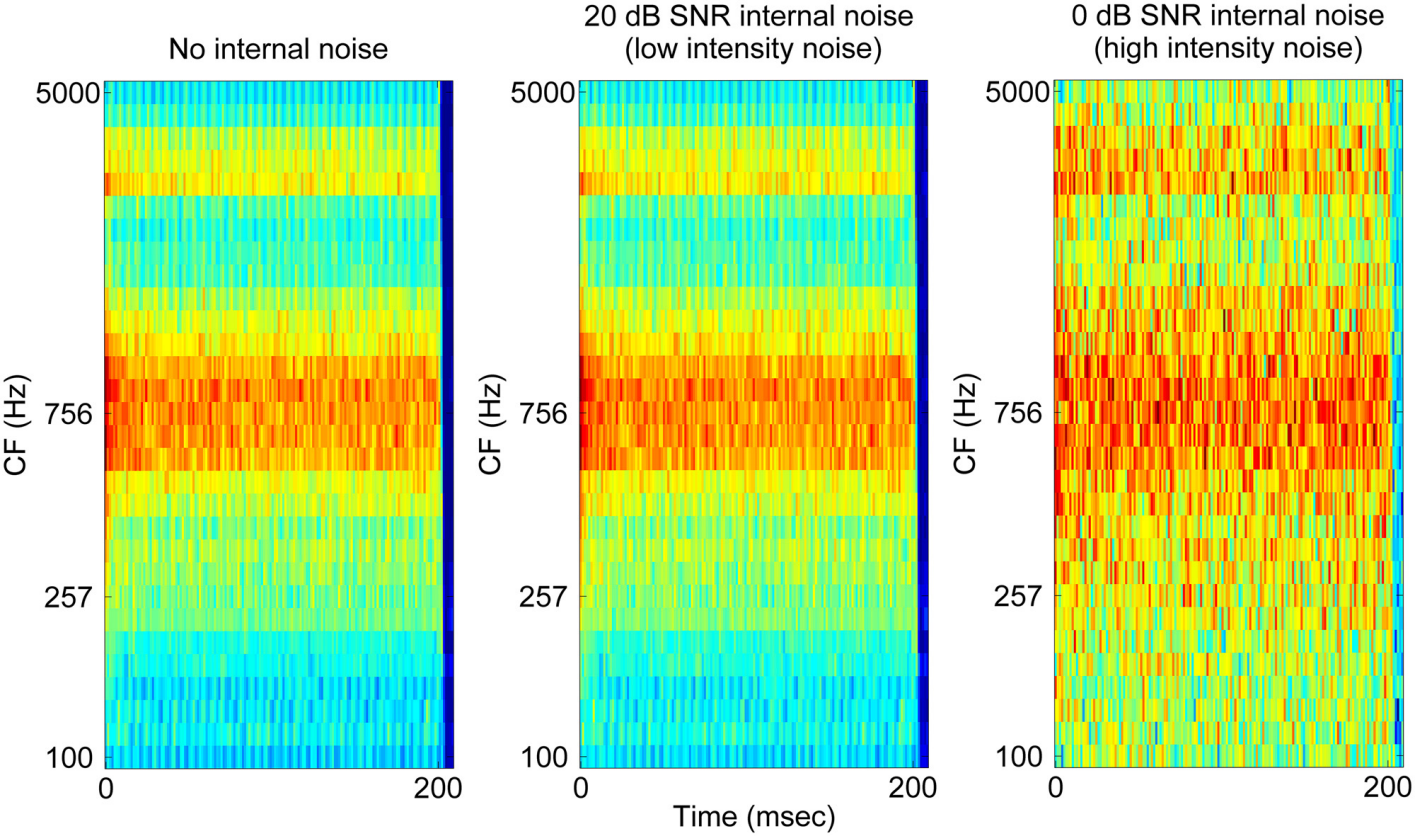
Effects of internal noise at the level of neurogram generation on the representations of the vowel /ɑ/ (S2 File) in the auditory-nerve neurograms.

**Fig 2:**
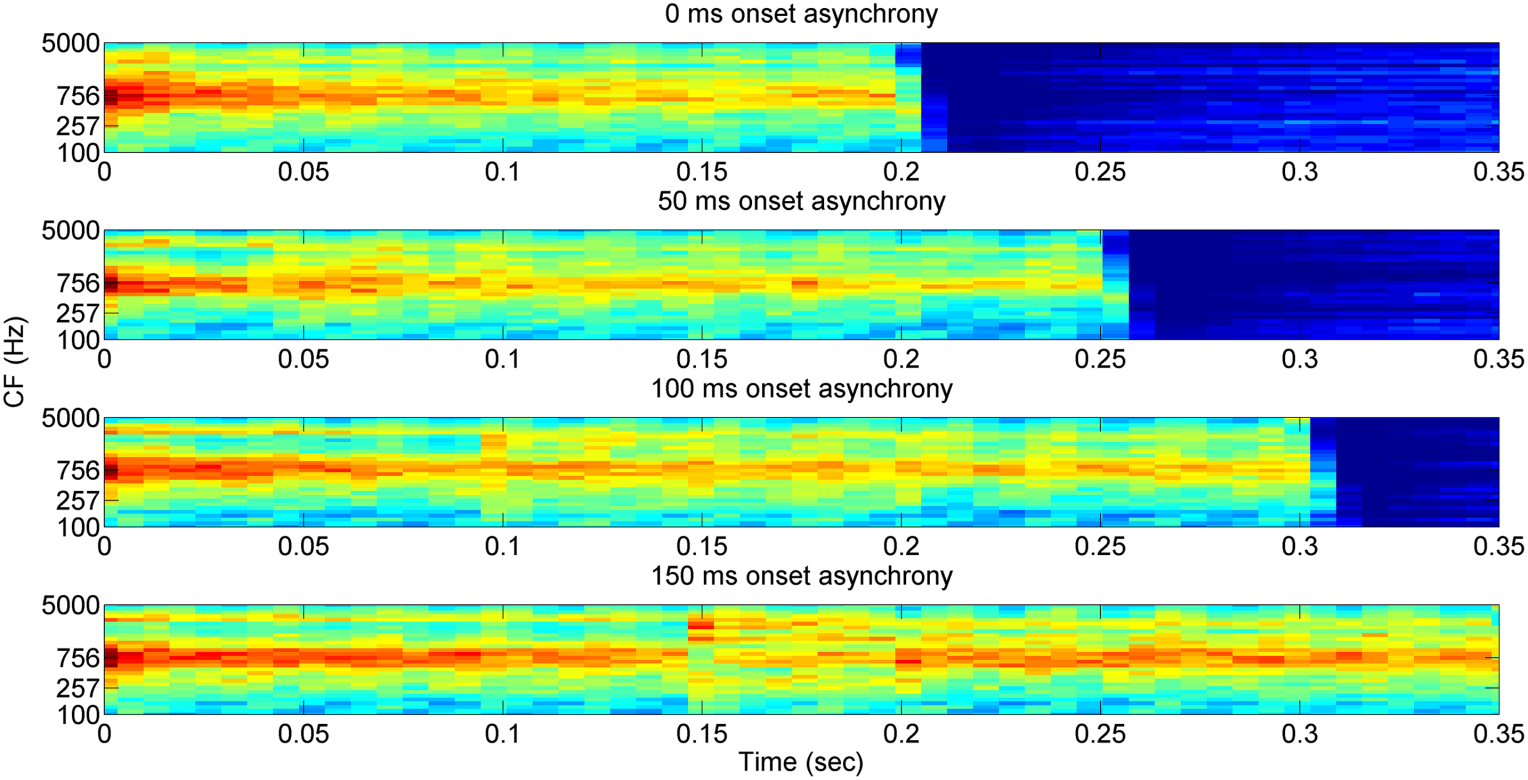
Neurograms for the vowel pair /ӕ ɑ/ with the onset asynchrony of 0, 50, 100, and 150 ms. For these neurograms, no internal noise was used.

If two completely different vowel stimuli are presented to human listeners, the auditory system would produce very different neural representations of the two vowels, resulting in different percepts of the two vowels. In order to assess different patterns of neural representations of vowels, an objective quantification method is required. For this purpose, we employed the “Neurogram Similarity Index Measure” (NSIM) [29, 30] to evaluate similarity between the neural representations of two different stimuli. Here, neurograms refer to a graphical representation of the neural discharge patterns of AN fibers, where discharge rate information for auditory channels is plotted over the duration of the acoustic stimulus. In this study, neurograms were constructed in the form of 30-by-38 matrices (i.e., 30 CFs in the ordinate and 38 time bins in the abscissa) based on the PSTH information simulated with the AN model [23]. In Fig 1(A), a neurogram for the vowel /ɑ/ is shown. In this neurogram, different strengths of neural activity were depicted as a color-scale from 0 (blue) to 190 spikes/s (red) of dynamic range for each frequency channel (along the vertical axis) and over time (along the horizontal axis). In comparison, Fig 2 shows neurograms of the vowel pair /ӕ ɑ/ with the onset asynchrony of 0, 50, 100, and 150 ms.

The NSIM index quantified the similarity between neural responses to different vowel stimuli. Briefly, for two different stimuli passed through the AN model, one served as a reference stimulus and the other served as a test stimulus. Using patches of pixels spanning the images of neurograms, the NSIM index between reference (“R”) and test (“T”) neurograms was computed as a weighted function of “luminance” (*μ*_*R*_*μ*_*T*_), “contrast” (*σ*_*R*_*σ*_*T*_), and “structure” (*σ*_*RT*_) statistics (in the form of 28-by-36 matrices), as follows:

$$NSIM=\;{\left(\frac{2\mu_{R}\mu_{T}+\;C_{1}}{\mu_{R}{}^{2}+\mu_{T}{}^{2}+C_{1}}\right)}^{\alpha }\times {\left(\frac{2\sigma_{R}\sigma_{T}+\;C_{2}}{\sigma_{R}{}^{2}+\sigma_{T}{}^{2}+C_{2}}\right)}^{\beta }\times {\left(\frac{\sigma_{RT}+\;C_{3}}{\sigma_{T}\sigma_{R}+C_{3}}\right)}^{\gamma }$$ (1),

in which μ and σ represent the mean and standard deviation of 3×3 square windows, which moves pixel by pixel over the entire image. The weighted contributions (α, β, γ) determined a single patch value. In accordance with [29], the optimally weighted functions were used (i.e. α = 1, β = 0, γ = 1) to obtain the simulation data in the current study. In Eq (1), *C1*, *C2* and *C3* are constants, used to prevent instability when values are small. Previous work [31], however, noted that C1, C2, and C3 had negligible influence on the image comparison result. An overall NSIM index was computed by averaging the NSIM values over time and 30 CFs. An NSIM index of 1 implies that two neurograms were perfectly correlated in neural information. Conversely, an NSIM index of 0 implies that two neurograms were uncorrelated, which means that two stimuli produced uncorrelated neural information at the level of the AN.

#### Computational model predictions of concurrent vowel identification

**Fig 3:**
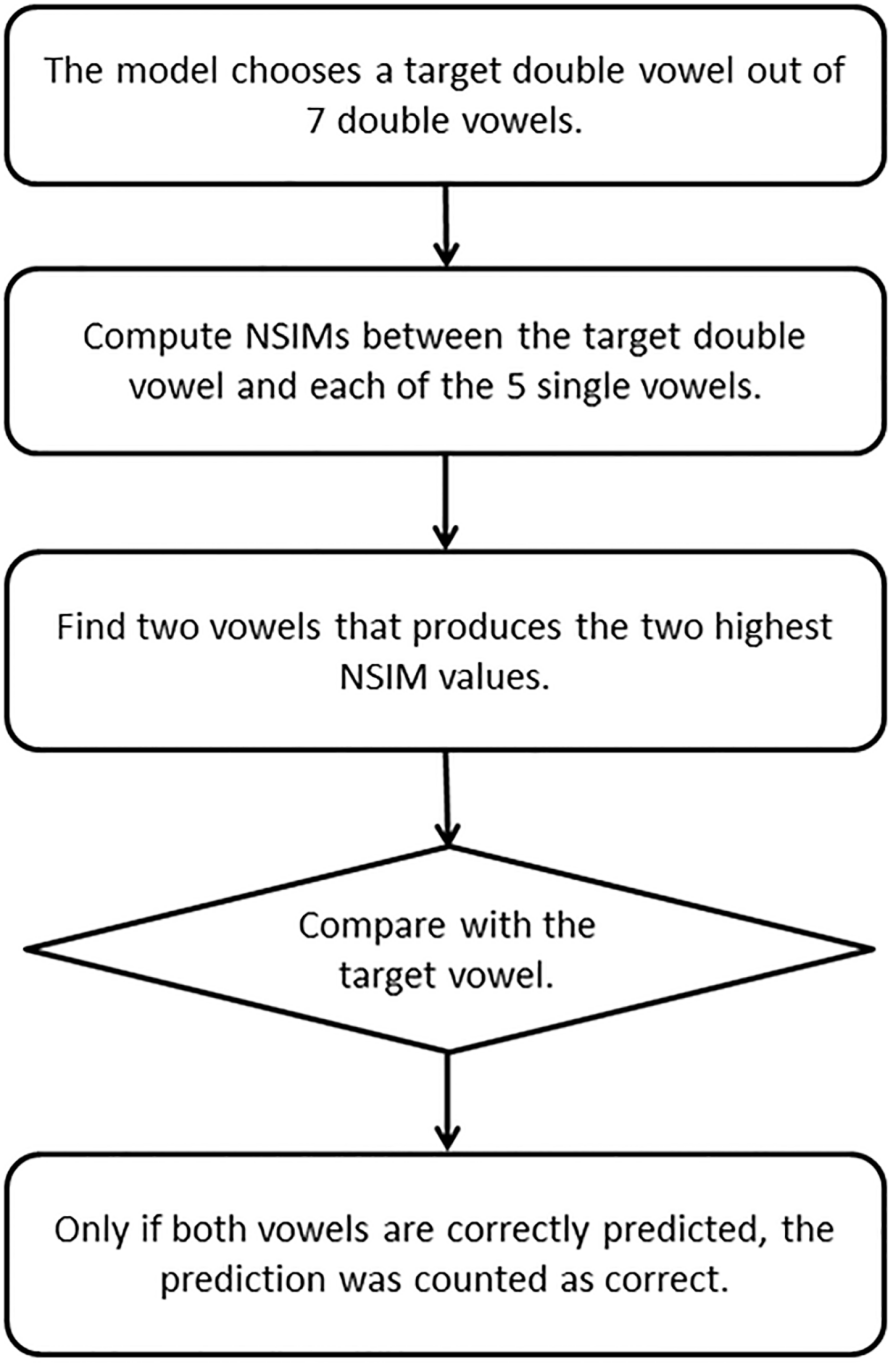
Diagram of the model implementation that predicts concurrent vowel identification using the AN responses.

To gain further insight on how human listeners would utilize the AN neural discharge information for concurrent vowel identification, the concurrent vowel identification performance for human subjects was simulated based on the AN discharge patterns. This computational model prediction of concurrent vowel identification was performed using the exactly same testing paradigm and stimuli as with human subjects. Fig 3 shows the diagram of the model implementation. In each trial, the model testing program randomly selected a “target” pair of concurrent vowels out of seven pairs, and the AN model outputs were then compared between the target pair of concurrent vowels and each of the five single vowels. The single vowel was provided to the AN model one by one. Therefore, to make a single decision, five different NSIM indices were computed.

The above procedure was performed independently for all the onset asynchrony values to compare the predicted concurrent vowel identification scores. The decision model had a two-step process. First, 5 different NSIM indices (for 5 single vowels) were scanned to determine the two single vowels that produced the first and second highest NSIM indices to the target pair. Second, the model testing program compared the predicted vowels with the original target pair. When the model correctly predicted both vowels, the testing program recorded the neural-observer’s response as correct. Per each testing run, each of the seven target vowel pairs was presented to the model 5 times in random order and a percent correct score was thus calculated after 35 vowel-pair presentations as the percent of both vowels correctly predicted by the model. For each testing condition, the mean prediction score averaged across 14 model runs was obtained.

#### Implementation of the internal noise

**Fig 4:**
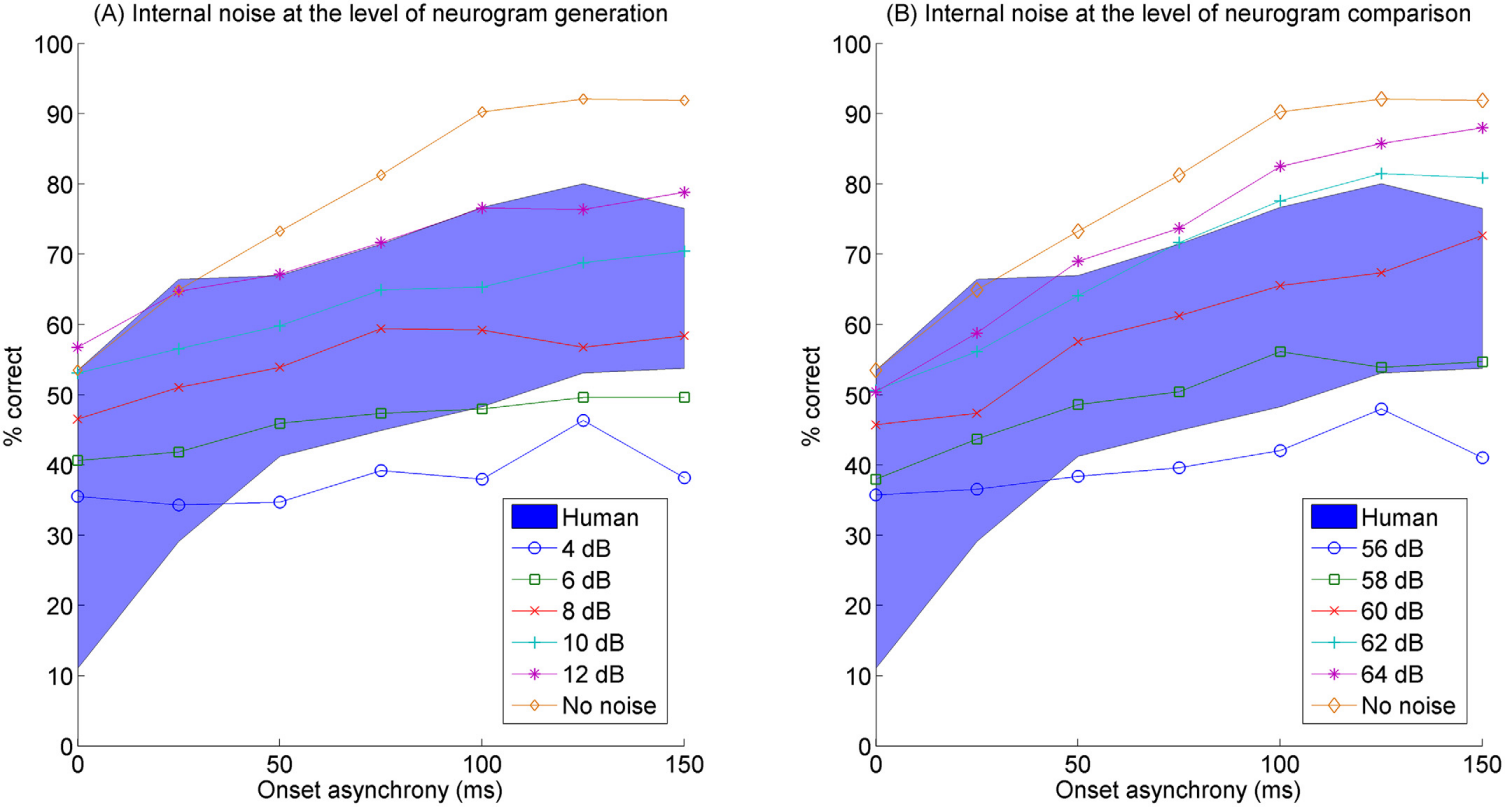
Predicted concurrent vowel identification scores by the computational model as a function of onset asynchrony. The range of concurrent vowel identification scores (mean ± one standard deviation) obtained from 14 human subjects in Hedrick and Madix (2009) is shown as a blue-colored area. Model predictions in the current study are shown for the internal noise at the level of neurogram generation (A) and comparison (B). Note that a different range of the level of internal noise was used for the left and right panels (see legends). Error bars for the model predictions are not plotted for clarity, but standard errors across 14 model runs were generally below 3%.

Without any internal noise, the model predictions of concurrent vowel identification showed better identification performance than human subjects (see Fig 4), because the model did not take into account any effect of trial-to-trial variability on predicted performance, which is widely observed in neural systems [32, 33]. Such trial-to-trial variability would obscure the pattern of neural dynamics of a single-trial neural response to an acoustic stimulus [34], thereby decreasing performance in model predictions. In the current study, internal noise was implemented either at the level of the neurogram generation or at the neurogram comparison level. Acoustic signals present well-defined speech information when they arrive at the listener’s ear, but the signals are then transformed into mechanical energy in the middle ear. The cochlea transforms the original acoustic information into the discharge patterns of the AN responses. Due to cochlear nonlinearities, the AN neural information might be delayed, distorted, or influenced by descending efferent neural activity. Internal noise may also be generated by the stochastic nature of neuronal firing or from different levels of arousal or fluctuations in attention [35, 36, 37]. We sought to determine if adding internal noise to either the neurogram generation or neurogram comparison schemes would show the best fit to the human data.

To simulate internal noise affecting neurogram generation, a Gaussian noise matrix was added to an original neurogram. A Gaussian noise was selected because it is simple (probability density function equal to a normal distribution) and is frequently used in studies examining sensory noise [38, 39]. The Gaussian noise matrix was created newly for each simulation (i.e. fresh noise) with the same size as the original neurogram (30-by-38 matrix). The strength of the internal noise was controlled by varying the signal-to-noise (SNR) between the RMS values of the original neurogram matrix and the Gaussian noise matrix. In Fig 1(A), the neurogram for the vowel /ɑ/ in the absence of any internal noise is shown, whereas Fig 1(B) and 1(C) shows the neurograms for the same vowel with increasing levels of internal noise (20 and 0 dB SNR, respectively). Note that the higher SNR values indicate the low intensity internal noise. When internal noise registering a 20 dB SNR was added to the original neurogram, the neural activities were generally well preserved (Fig 1B). However, when the strength of the internal noise was increased to 0 dB SNR, the neural activities associated with formant frequencies were greatly obscured (Fig 1C).

To simulate the internal noise affecting neurogram comparison procedures, Gaussian noise matrices (size: 28-by-36) were added to the matrices of the luminance and structure statistics in Eq 1 to introduce probabilistic decision behavior. The strength of the internal noise here was varied by changing the SNR between the RMS values of the luminance / structure statistics and the Gaussian noise matrix. To quantify the difference between the human data and model predictions, the mean squared error (MSE) was computed as a function of the strength of internal noise as follows:

$$MSE=\frac{1}{N}\overset{N}{\underset{i=1}{\sum }}{\left(Observed\;score_{i}-Predicted\;score_{i}\right)}^{2}$$ (2)

## Results

Fig 4 shows mean identification scores averaged across 14 human subjects and across 14 model runs as a function of onset asynchrony. The blue colored areas show the range of identification performance (mean ± one standard deviation) for 14 human NH subjects from the previous study [12]. In Fig 4, different symbols represent different model conditions. Error bars for the model predictions were not plotted for clarity, but standard errors across 14 model runs were generally below 3 percentage points. Identification performance increased with increasing onset asynchrony in both human subjects and the neural-observer model.

Fig 4(A) shows model predictions with the internal noise at the level of neurogram generation for noise levels between 4 and 12 dB SNR. Likewise, Fig 4(B) shows model predictions with the internal noise affecting neurogram comparisons, but note that different levels of internal noise was used here: 56 to 64 dB SNR. Table 2 shows the mean square error (MSE) for the internal noise at neurogram generation or neurogram comparison as a function of the level of internal noise. The MSE results demonstrated that, for the internal noise affecting neurogram generation, the lowest MSE was shown for an 8 dB SNR. In contrast, for the internal noise affecting neurogram comparison, the lowest MSE was found for a 60 dB SNR. Therefore, much more internal noise was required to simulate the human data when the internal noise was introduced at the level of neurogram generation.

**Table 2:** The mean square error (MSE) between the human data and model predictions as a function of the level of the internal noise. The best match between the human and model data was observed at 8 and 60 dB signal-to-noise ratio (SNR) for the internal noise simulated at the level of neurogram generations and comparisons, respectively.

(A) Internal noise simulated at the level of neurogram generations						
SNR	4 dB	6 dB	8 dB	10 dB	12 dB	No noise
MSE	380.2	146.6	52.6	90.0	245.8	540.1
(B) Internal noise simulated at the level of neurogram comparisons						
SNR	56 dB	58 dB	60 dB	62 dB	64 dB	No noise
MSE	295.8	64.0	38.6	198.0	315.0	540.1

**Fig 5:**
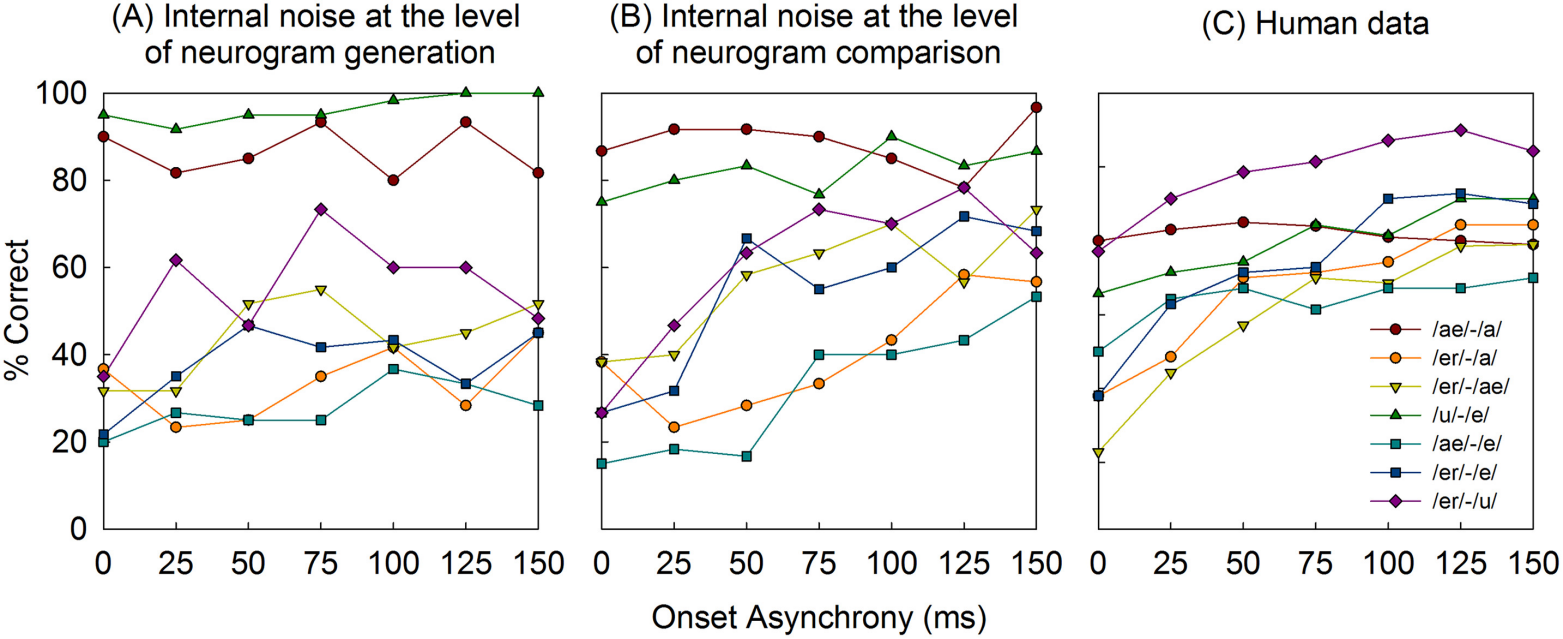
Predicted concurrent vowel identification scores as a function of onset asynchrony, with the legend showing the seven individual vowel pairs. Model predictions are shown for the internal noise at the level of neurogram generation (A) and comparison (B). Human data reported from Hedrick and Madix (2009) are replotted in (C).

In Fig 4 the range of human identification performance is rather large across different vowel pairs. By collapsing data across vowel pairs, information is necessarily lost. Therefore, Fig 5(A) and 5(B) shows model predictions for individual vowel pairs for the SNR conditions showing the best match to the human data. To facilitate the comparison between the model predictions and human data for individual vowel pairs, the human data reported in Figure 1 from Hedrick and Madix (2009) are also plotted in Fig 5(C). Note in particular that the model predictions with internal noise at the level of neurogram comparison show slopes similar to that of the human data from Hedrick and Madix (2009) [12], whereas model predictions with internal noise at the level of neurogram generation all show shallow slopes with increasing onset asynchrony.

## Discussion

This paper presents a neural computational modeling study of concurrent vowel perception. Previous research has shown that concurrent vowel perception improves with increasing temporal onset asynchrony of the vowels, even if the vowels have the same fundamental frequency. The current study investigated possible underlying neural processing factors involved in concurrent vowel perception. The individual vowel stimuli from a previous study were used as inputs for a phenomenological AN model. Resultant neurograms of concurrent vowel stimuli were then matched with the neurograms of the single vowels using a quantitative comparison metric (NSIM). To facilitate optimum matches between the model data and the behavioral data, internal noise was added at either an early stage of processing (to the neurogram) or a late stage of processing (to the NSIM procedure). Our results show that neurograms derived from a phenomenological AN model are quite good at distinguishing concurrent vowels, and that noise has to be added to help the model match data from listeners with NH. The best fit to the behavioral data was achieved with a SNR of 8 dB for noise added at the level of neurogram generation, and with a SNR of 60 dB for noise added at neurogram comparison. Thus, a substantial amount of noise had to be added at the level of neurogram generation to match the human data; this result reflects the apparent robustness of encoding of acoustic information by the peripheral auditory system. Only a small amount of noise, however, had to be added to the neurogram comparison procedure to match the human data. Although we did not manipulate F0 nor attempt to simulate F0 cues in the current study, our findings are in general agreement with recent work that has shown that the accepted model for concurrent vowel perception, relying on F0 segregation, is incomplete and that other factors are at play [22].

The current study presents a unique theoretical framework for concurrent vowel identification that provides a link between the acoustic temporal waveform and its neural representation in the AN fibers. Furthermore, the link between peripheral and central processing of concurrent vowel identification was modeled. The predicted concurrent vowel identification data suggest that the implementation of internal noise is critical to accurately simulate the human data. The significant role of internal noise in the current study is consistent with the findings from other sensory systems such as the visual system [40], the somatosensory system [41], taste processing [42], and with general cortical functions [43].

The relative difference in the effectiveness of the internal noise at the level of neurogram generation and neurogram comparison suggests that different processes or stages in concurrent vowel identification may thus be affected differently by internal noise effects. The use of internal noise may reflect processes that are not included in AN response neural modeling, and may more likely arise from neural properties or from attention or arousal mechanisms. There was no attempt in the current study to model attention or arousal, nor to model components of working memory such as the phonological loop [44] or template matching [13]. Nevertheless, the preliminary nature of the current study does lend itself to further explorations of these components, as well as to explore why internal noise had such differing effects at different modeling levels. The current study demonstrated that there was a difference in the strength of internal noise at the levels of neurogram generations and comparisons that produced the best account of the human data. A smaller amount of the internal noise (60 dB SNR) was required at the level of neurogram comparisons to produce the best match with the human data, including creating slopes similar to that of human data, whereas relatively strong internal noise was needed at the level of neurogram generations (8 dB SNR) to simulate the human data. These simulation data suggest the importance of the category assignment and decision-making process for concurrent vowel identification that may occur in more central auditory system beyond the cochlear nerve. In addition, human listeners’ psychoacoustic performance could be substantially affected by the central “processing efficiency”, which is the listener’s ability to use optimally the peripheral information [45, 46, 47]. We speculate that the variability in concurrent vowel identification performance might be partly related to differences in the central “processing efficiency” across individual subjects. Such hypothesis warrants a future investigation.

Of particular interest is extension of the psychoacoustic and computational modeling framework to other acoustic parameters (e.g., F0, stimulus levels, and larger segments of speech than vowels) and biological parameters (e.g., effects of sensorineural hearing loss, aging). A recent study [48] demonstrated that the neuronal populations in primary auditory cortex of monkeys can resolve the lower harmonics of single harmonic complex tones via the rate-place code. More importantly in relation to the current study, it was recently demonstrated that the primary auditory cortex represents the lower harmonics and formant information in a sufficient manner to drive concurrent vowel identification [49]. Thus it may be plausible to speculate that the auditory peripheral processing is necessary but not sufficient in terms of segregating vowels, and the role of the central processing may be more critical for the optimum vowel identification process.

Extension of the current work to elderly listeners and to listeners with sensorineural hearing loss could, to some degree, determine the effect size of peripheral and more central processing in perception of concurrent vowels. Based on information processing models of speech perception [50, 51], the interaction of peripheral and more central processing may not allow for a completely clear separation of lower and upper levels of processing, in contrast to models of a serial, hierarchical nature [52]. There is yet insufficient information on how efferent auditory processing might influence concurrent vowel perception [21, 53, 54]. Extensions of the current study to include participants who have slower cognition and/or a peripheral hearing loss, or who use cochlear implants could thus provide valuable theoretical and clinical information.

It may be that onset asynchrony benefit was realized in listeners by simply attending to portions of the asynchronous pairs in which only one vowel was present. Because the current study does not present behavioral data for different time slices of the asynchronous pairs, nor does our model examine different time slices of the asynchronous pairs, we cannot say with certainty that improvements in vowel identification with increasing onset asynchrony relies solely on higher-order non-primitive mechanisms. This will remain an issue for further work.

## Supporting Information

S1 File Single vowel /e/. (WAV)

S2 File Single vowel /a/. (WAV)

S3 File Single vowel /u/. (WAV)

S4 File Single vowel /ae/. (WAV)

S5 File Single vowel /er/. (WAV)
